# Enhancing handwritten text recognition accuracy with gated mechanisms

**DOI:** 10.1038/s41598-024-67738-8

**Published:** 2024-07-22

**Authors:** Ravikumar Chinthaginjala, C. Dhanamjayulu, Tai-hoon Kim, Suhaib Ahmed, Si-Yeong Kim, A. S. Kumar, Visalakshi Annepu, Shafiq Ahmad

**Affiliations:** 1 School of Electronics Engineering, Vellore Institute of Technology, Vellore, India; 2 School of Electrical Engineering, Vellore Institute of Technology, Vellore, India; 3 School of Electrical and Computer Engineering, Yeosu Campus, Chonnam National University, 50 Daehak-Ro, Yeosu-Si, Jeollanam-Do 59626 Republic of Korea; 4 Department of Electronics and Communication Engineering, Model Institute of Engineering and Technology, Jammu, J&K India; 5 Bluecrest University, 1000 Monrovia, Liberia; 6 School of Computer Science and Engineering, VIT-AP University, Amaravati, 522 237 India; 7 Industrial Engineering Department, College of Engineering, King Saud University, P.O. Box 800, Riyadh, 11421 Saudi Arabia

**Keywords:** Convolutional recurrent neural networks, Handwritten transcript recognition, Natural language processing, Gated convolutional neural networks, Deep learning, Biomedical engineering, Electrical and electronic engineering

## Abstract

Handwritten Text Recognition (HTR) is a challenging task due to the complex structures and variations present in handwritten text. In recent years, the application of gated mechanisms, such as Long Short-Term Memory (LSTM) networks, has brought significant advancements to HTR systems. This paper presents an overview of HTR using a gated mechanism and highlights its novelty and advantages. The gated mechanism enables the model to capture long-term dependencies, retain relevant context, handle variable length sequences, mitigate error propagation, and adapt to contextual variations. The pipeline involves preprocessing the handwritten text images, extracting features, modeling the sequential dependencies using the gated mechanism, and decoding the output into readable text. The training process utilizes annotated datasets and optimization techniques to minimize transcription discrepancies. HTR using a gated mechanism has found applications in digitizing historical documents, automatic form processing, and real-time transcription. The results show improved accuracy and robustness compared to traditional HTR approaches. The advancements in HTR using a gated mechanism open up new possibilities for effectively recognizing and transcribing handwritten text in various domains. This research does a better job than the most recent iteration of the HTR system when compared to five different handwritten datasets (Washington, Saint Gall, RIMES, Bentham and IAM). Smartphones and robots are examples of low-cost computing devices that can benefit from this research.

## Introduction

The field of handwritten text recognition (HTR) has many uses in both the academic and professional worlds. By using either a static or dynamic information mode, the HTR changes the handwritten text to numeric codes (ASCII or Unicode)^1^. Images can therefore be thought of as the data for offline text recognition, which can then assist in digitizing scripts^2^, medicinal archives^3^, solicitations^4^, and various other types of documents. These programs promote the growth of HTR for various scripts and languages.

The off HTR was originally intended to be used for sequence matching, which involves simulating features taken from input images and arranging them into a sequence with an amount produced order that guides this one to a grouping of characters. Primarily, the Hidden Markov Model (HMM) was the strategy that proved to be most effective in resolving the HTR issue^5^. But because Markov made the supposition that for each statement depends only on its present state, the model was impotent to make use of setting data.

The research in HTR over the last few years has demonstrated significant improvements over HMM. Convolutional—Recurrent Neural Networks (C-RNN) are a nature of deep learning technique that has undergone significant advancement and produced useful outcomes in industrial applications^6^. The C-RNN model uses Long Short-Term Memory as a sequence decoder^7^. By incorporating multi-dimensional data into the RNN architecture, the Multi-dimensional LSTM (MDLSTM)^8^ is used to increase the accuracy of HTR. Due to MDLSTM's high complexity and computational expense, the most recent studies for the HTR problem yield bidirectional LSTM (BLSTM)^9^ results. With less computational complexity and expense, the BLSTM provides comparable results to the MDLSTM.

The vanishing gradient problem makes it difficult for models using BLSTM, like CNN-BLSTM, to remember extensive contexts even though they produce excellent results. Additionally, the high parameter requirements of the current optical models necessitate a large amount of trainable data. It poses a significant challenge for applications in the real world^10^. The Gated-CNN-BLSTM technique is recycled to decrease the factors in order to address the problem of a huge number of factors but would have an impact on the model's performance^11^.

We employ the Gated-Convolutional Recurrent Neural Network (Gate-CRNN) architecture, which makes use of a gated mechanism developed by Dauphin^12^, to improve the accuracy of the offline HTR systems. A bidirectional gated recurrent unit is also included in the model (BGRU). In order to achieve higher accuracy, the suggested optical model, Gated-CNN-BGRU, would need smaller quantity factors.

### Problem formulation

When it comes to issue formulation in "Enhancing Handwritten Text Recognition Accuracy with Gated Mechanisms," the primary focus is on incorporating gated mechanisms to improve the accuracy of handwritten text recognition systems. The primary focus of this study is on the difficulties that emerge from distracting and uneven handwriting styles, which frequently result in errors in recognition assessments. The researchers are optimistic that by introducing gating mechanisms into the recognition process, they will be able to improve the model's accuracy by increasing its ability to collect meaningful data and context from handwritten inputs. This concept provides a framework for determining how effective gated mechanisms are at improving the performance of handwritten text recognition. This is done to meet the present concerns in the field.

### Motivation

**"**Enhancing Handwritten Text Recognition Accuracy with Gated Mechanisms" was developed as a solution to the age-old problem of accurately transcribing handwritten text, especially in contexts with high noise levels and a wide range of handwriting styles. Traditional approaches are sometimes unable to capture the myriad intricacies of handwritten input, resulting in poor performance and lower usability in real-world applications such as document digitalization and text analysis. The scientists want to improve the accuracy and resilience of handwritten text recognition systems by utilising gated mechanisms, which have shown effective in gathering contextual information and long-range interactions in a variety of machine learning applications. This will be performed using gated techniques. To be fully effective, automatic text transcription systems require major improvements in recognition algorithms that can accommodate a wide range of handwriting styles.

The following are the key aids through this reading:

- The results based on CNN-BLSTM are enhanced by a new architecture called Gated-CNN-BGRU.
- Allowing you to adapt to various noises, styles, and variations with a smaller amount of practice data.
- The number of parameters is reduced in comparison to the conventional model when using the Gated-CNN-BGRU prototypical to diminish calculation costs and shrink the model (CNN-BLSTM).

Washington, Bentham, RIMES, Saint Gall, and IAM are the five well-known datasets used to train and investigate the suggested model^15^. After that, the output of the suggested models is contrasted with that of Puigcerver^13^, Flor^18^, and Bluche^19^.

The design complexity of deep neural networks is another crucial factor to consider when comparing them, since it affects both how much space the model occupies and how long it takes to decode data. When seen in this light, the suggested model is comparable to the Bluche model in terms of the number of trainable parameters (thousands), and is a significant reduction from Puigcerver's model (millions). We took the average of each optical model throughout all the different rounds of the experiment to calculate the decoding time. As a result, the model that was proposed was the one that fell somewhere in the middle, with the Bluche model being the one that was the quickest and Puigcerver being the one that was the slowest.

The remaining paper is planned as given below:

- Literature analysis of Puigcerver, Bluche, and Flor models in Section "Related works".
- In sector 3, the suggested model and parameter changes are described.
- Datasets and methodology are labelled in Section "Datasets and methods".
- The results and summary are deliberated for each dataset differently in segment 5

## Related works

In this paper, HTR systems follow the given steps:

- Inputs for CNN layers are in form of images which results in features.
- The features extracted from CNN are mapped in both directions of the sequence through the RNN layers.
- Lastly, to decode the output text for inference and to calculate the loss values using Connectionist Temporal Classification (CTC)^16^.

Traditionally, HTR systems have been conceived of as problems involving sequence matching. In this model, a sequence of features gleaned from the input data is compared and contrasted with a sequence of text characters that constitutes the output. First, a segmentation and graph search method were used to complete the transcribing assignment. Next, Hidden Markov Models (HNMs) were utilized. HMMs are unable to utilize the context information contained in a text sequence, because they are based on the Markovian assumption that every observation depends only on the present state.

In order to circumvent HMM limitations throughout the past few years, CNNs have been used. Also, due to their minimal complexity and excellent performance, the BLSTM layers have also been employed frequently for the propagation of features. Finally, the CTC receives the output of the recurrent layers, decodes it into final text, and uses it to calculate the loss value (training mode). In this scenario, more straightforward optical models were devised in order to achieve the same or better performance as their more conventional counterparts.

Therefore, Puigcerver^13^ suggested the CNN-BLSTM architecture as a means of reducing the computational cost while simultaneously achieving better results than cutting-edge models that utilized Multidimensional LSTM (MDLSTM) layers. This was done in order to achieve better results. In a similar vein, Bluche et al. presented an architecture called Gated-CNNBLSTM. In order to extract more useful information, this design uses the Gated technique in the convolutional layers. As a result, the optical model can use a lot less factors while still producing outstanding results.

Finally, Neto et al^19^. showed that the usage of the Gated-CNN-BGRU architecture improved the HTR area. To extract more useful features, this architecture employs the BGRU in the recurrent layers and the Gated mechanism in the convolutional layers. As a result, a novel optical model was introduced that performed superbly in terms of recognition even when there was a small amount of data to work with.

Kumari et al^38^. addressed the specified demand. Improved models for handwritten text recognition rely heavily on adaptive feature selection, which is made possible by the gating mechanism, which controls the flow of input. This process is responsible for enabling this progress. In addition, the attention module supports internal line segmentation, allowing pages to be processed line by line. This is enabled via the attention module. After the decoding phase, a post-processing step is performed with a word beam search decoder based on connectionist temporal categorization. Our approach builds on LexiconNet's existing architecture by carefully incorporating gated convolutional layers into the deep neural network. The character error rates for the IAM, RIMES, and READ-16 datasets were 2.27%, 0.9%, and 2.13%, respectively. On the other hand, the word error rates on the IAM, RIMES, and READ-2016 datasets were 5.73%, 2.76%, and 6.52%, respectively, suggesting that the proposed GatedLexiconNet outperforms at both the line and paragraph levels.

Omidi et al^39^. proposed an end-to-end neural architecture for HDSR that is data efficient and built on the HTR workflow. The architecture is a recurrent connection-free gated fully convolutional network that was trained using CTC loss functions and then improved using two augmentation methods. Our top recognition rates were 95.41%, 95.90%, and 88.06% on the ORAND CAR-A, ORAND CAR-B, and CVL datasets, respectively, when we used ICFHR 2014 competition measures.

Qu et al^40^. provided a novel end-to-end attention convolutional recurrent network (EACRN) for online handwritten Chinese text recognition (OHCTR). The EACRN architecture uses a CNN to extract local contextual characteristics from raw sequential coordinates, followed by bidirectional Long Short-Term Memory (BiLSTM) layers that capture long-term dependencies. Multiple-head attention mechanisms are then used to weigh these local contextual features. In addition, we offer a focal Connectionist Temporal Classification (CTC) objective function to direct attention to low-frequency features and increase prediction accuracy. Experimental evaluations on two publicly available datasets, the CASIA-OLHWDB2.0–2.2 benchmark and the IAHCT-UCAS2018 in-air handwritten Chinese text dataset, show that our approach achieves higher recognition accuracy, faster computation speed, and a more compact model than previous CNN architectures.

Alshawi et al^41^. created a collection of 20,000 images of Persian numerals, purposely include a wide range of problems to suit text recognition applications. In addition, we present a convolutional-based model that combines the squeeze and excitation gate mechanisms to emphasise latent characteristics, as well as connectionist temporal classification for end-to-end sequence learning in Persian digit identification. We thoroughly evaluate our suggested model against numerous architectures and models in order to determine its overall performance. As a result, our solution achieves an accuracy of 94.26 on our datasets, demonstrating its superiority over alternative methods and highlighting its effectiveness in Persian digit recognition.

In a comparative assessment of advances in Handwritten Text Recognition (HTR), Kumari et al. proposed a unique strategy based on gated mechanisms that demonstrated better accuracy. Their strategy is based on adaptive feature selection facilitated by gating devices, which controls input flow and improves progress. Furthermore, the addition of an attention module improves internal line segmentation, allowing for more efficient page processing. Using LexiconNet's architecture, they carefully added gated convolutional layers, resulting in greater performance with lower letter and word error rates across multiple datasets. Omidi et al. presented a data-efficient end-to-end neural architecture for Handwritten Digit String Recognition (HDSR), which achieved good recognition rates on benchmark datasets. Meanwhile, Qu et al. proposed an attention convolutional recurrent network (EACRN) specifically designed for online handwritten Chinese text recognition, exceeding earlier CNN architectures in terms of accuracy, speed, and model compactness. Finally, Alshawi et al. compiled a dataset of Persian numbers and developed a convolutional-based model for Persian digit recognition, demonstrating its usefulness and adding to the evolving ecosystem of HTR technology.

The well-known models are described in the below subdivisions.

### Convolutional recurrent neural network

**Figure 1:**
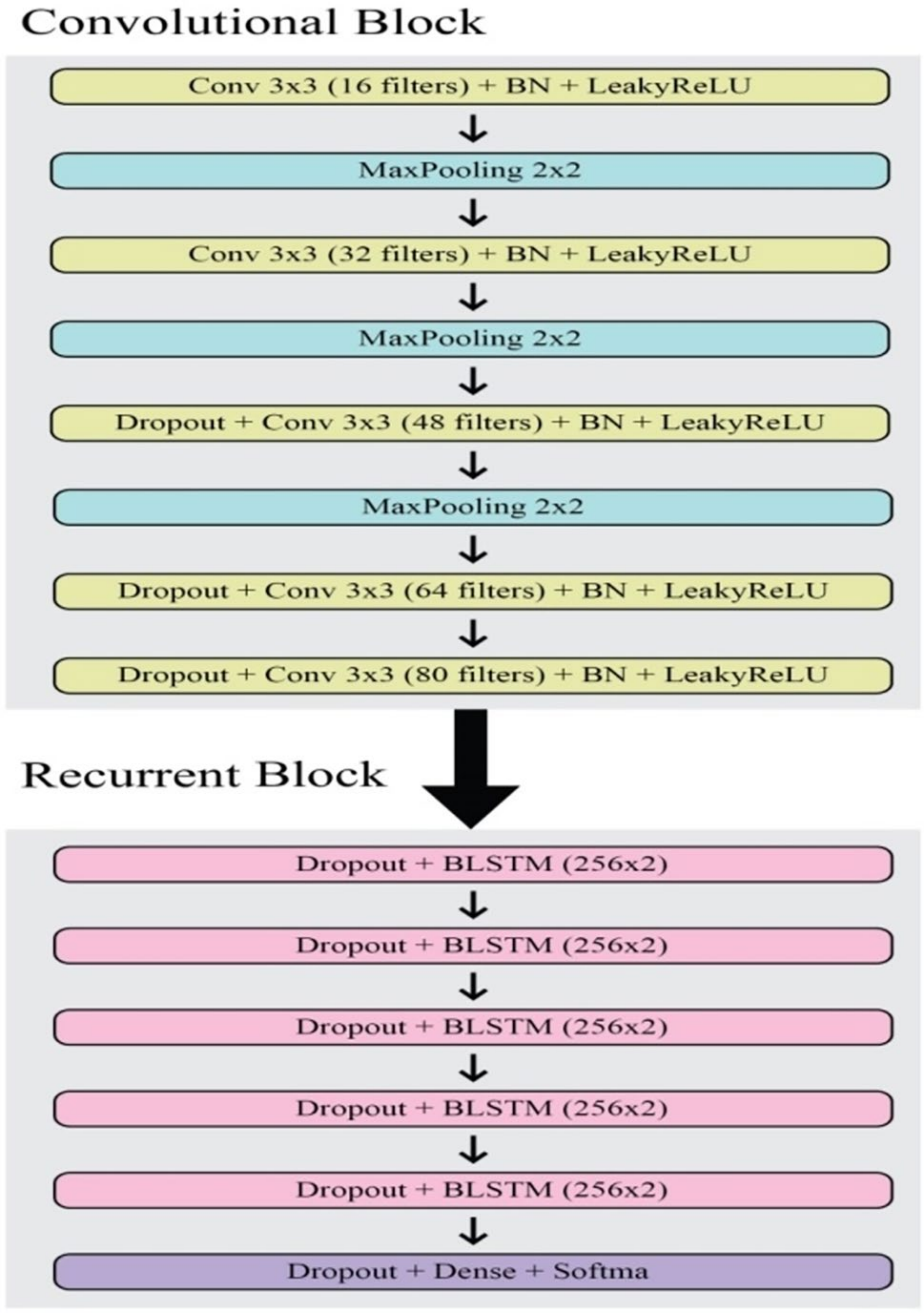
Puigcerver architecture.

The traditional CRNN approach has been introduced by Puigcerver^13^ which presents a high recognition rate but uses many parameters (approx. 9.6 million). Figure 1 depicts the architecture presented by Puigcerver which has five convolutional layers and five BLSTM layers in this architecture.

Figure 1 depicts the Puigcerver Architecture, an important model in the field of Handwritten Text Recognition (HTR). Puigcerver developed this architecture, which is often mentioned in the literature. It serves as a core framework for understanding and enhancing HTR technology. It consists of a number of finely built components and layers that process and analyse handwritten input, resulting in accurate recognition outcomes. The Puigcerver Architecture is often made up of convolutional neural network (CNN) layers for feature extraction, recurrent neural network (RNN) layers for sequential modelling, and attention mechanisms to focus on relevant information. This architectural design enables a comprehensive comprehension of handwritten text, resulting in robust recognition performance across a variety of datasets and applications. Furthermore, the Puigcerver Architecture is frequently used as a benchmark for assessing the efficacy of novel HTR models and methodologies, leading researchers in their efforts to improve recognition accuracy and efficiency.

The convolutional block consists of six parts i.e. (i) five convolutional layers of 3 × 3 kernels with an increasing number of filters per layer by a factor of 16 (16, 32, 48, 64, 80); (ii) Glorot uniform as an initializer^17^; (iii) Leaky Rectifier Linear Unit (LeakyReLU) as an activator^18^; (iv) Batch Normalization for nonlinear activation function^19^; (v) Maxpooling (2 × 2 kernels) for first three layers of the convolutional block; and (vi) Dropout (probability 0.2) for last three layers of the convolutional block. Dropout and Maxpooling are applied to overcome the problem of Overfitting^20^.

The recurrent block consists of three parts i.e. (i) five BLSTM layers with 256 hidden units each; (ii) a dense layer as the last layer of the recurrent block with a size of 1 (CTC null symbol) + charset size; and (iii) Dropout (probability 0.5) for all the layers of the recurrent block including the dense layer^20^.

### Gated Convolutional recurrent neural network

**Figure 2:**
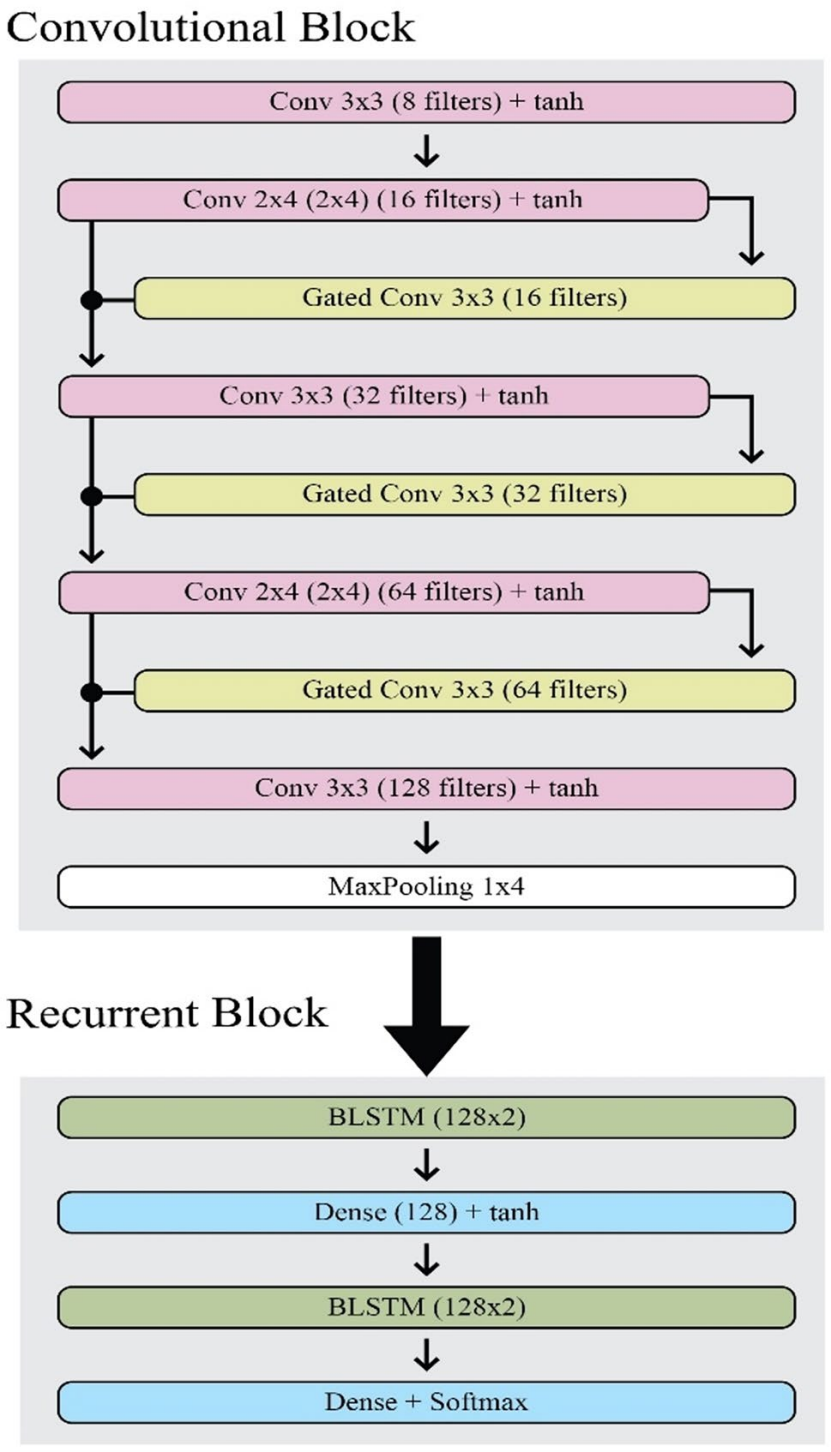
Bluche architecture.

The latest approach to Gated Convolution was introduced by Dauphin^12^. Bluche and Melina^14^ have used the concept presented by Dauphinto to produce a new architecture, Gated-CNN-BLSTM which brings out more significant features when compared with the CRNN architecture introduced by Puigcerver^13^. This approach also requires very few parameters (approx. 730,000) which results in a faster model. Figure 2 depicts the architecture presented by Bluche^14^, which includes 5 convolutional layers, 3 gated convolutional layers, and 2 BLSTM.

Figure 2 displays the Bluche Architecture, an important model in the field of Handwritten Text Recognition (HTR) established by Bluche that is well-known for its creative approach and performance. This architecture is precisely designed to solve the difficulties inherent in recognising handwritten text, particularly in situations when handwriting styles and noise levels vary significantly. The Bluche Architecture is often made up of interconnected modules that extract features from raw data, model sequential dependencies, and provide reliable text predictions. Key components may include convolutional neural network (CNN) layers for feature extraction, recurrent neural network (RNN) layers such as Long Short-Term Memory (LSTM) or Gated Recurrent Units (GRU) for sequential modelling, and attention mechanisms for dynamically focusing on relevant regions of the input. The Bluche Architecture achieves cutting-edge performance in handwritten text recognition tasks across a wide range of datasets and applications by leveraging these components synergistically. This design also acts as a standard for assessing the efficacy of new HTR procedures and techniques, giving vital insights and recommendations for future advances in the field.

The original piece (X) and the sigmoid initiation (S) of the unique piece, which are given, function as a point-wise product to create the gated mechanism^12^:

1 $${\text{Y }} = {\text{ S}}\left( {\text{X}} \right) \odot {\text{X}}$$

The convolutional block consists of 8 layers of which 5 are traditional ones and the remaining three are gated convolutional layers. The block can be divided into 6 parts i.e. (i) convolutional layer of 3 × 3 kernels with 8 filters; (ii) combination of gated convolutional layer of 3 × 3 kernels and a convolutional layer of 2 × 4 kernels with 16 filters each; (iii) combination of gated convolutional layer of 3 × 3 kernels and a convolutional layer of 3 × 3 kernels with 32 filters each; (iv) combination of gated convolutional layer of 3 × 3 kernels and a convolutional layer of 2 × 4 kernels with 64 filters each; (v) convolutional layer of 3 × 3 kernels with 128 filters; and (vi) to overcome overfitting Maxpooling is applied with 1 × 4 kernels. Similar to the Puigcerver model, Glorot uniform is used as the initiator. But the activator function is changed to hyperbolic tangent (tanh)^21^ in place of LeakyReLU.

The recurrent block consists of 4 layers i.e. (i) is a BLSTM layer with 128 hidden units; (ii) is a dense layer consisting of 128 hidden units with tanh as an activator; (iii) is a BLSTM layer with 128 hidden units; and (iv) is a dense layer as the last layer of the recurrent block with a size of 1 (CTC null symbol) + charset size.

### Gated convolutional neural network bidirectional gated recurrent unit

It is inspired from Puigcerver^13^ and Bluche^14^ models to aim for both better results and a low number of parameters respectively. The architecture was introduced by Flor. With a small difference in the formula, the gated contrivance is like the Bluche model. The original features are split in half, with the first half (H1) receiving the sigmoid function application and the other half (H2) receiving the pointwise product of the sigmoid function (S) and the first half (H1).

2 $${\text{Y }} = {\text{ S}}\left( {{\text{H1}}} \right) \odot {\text{H2}}$$

**Figure 3:**
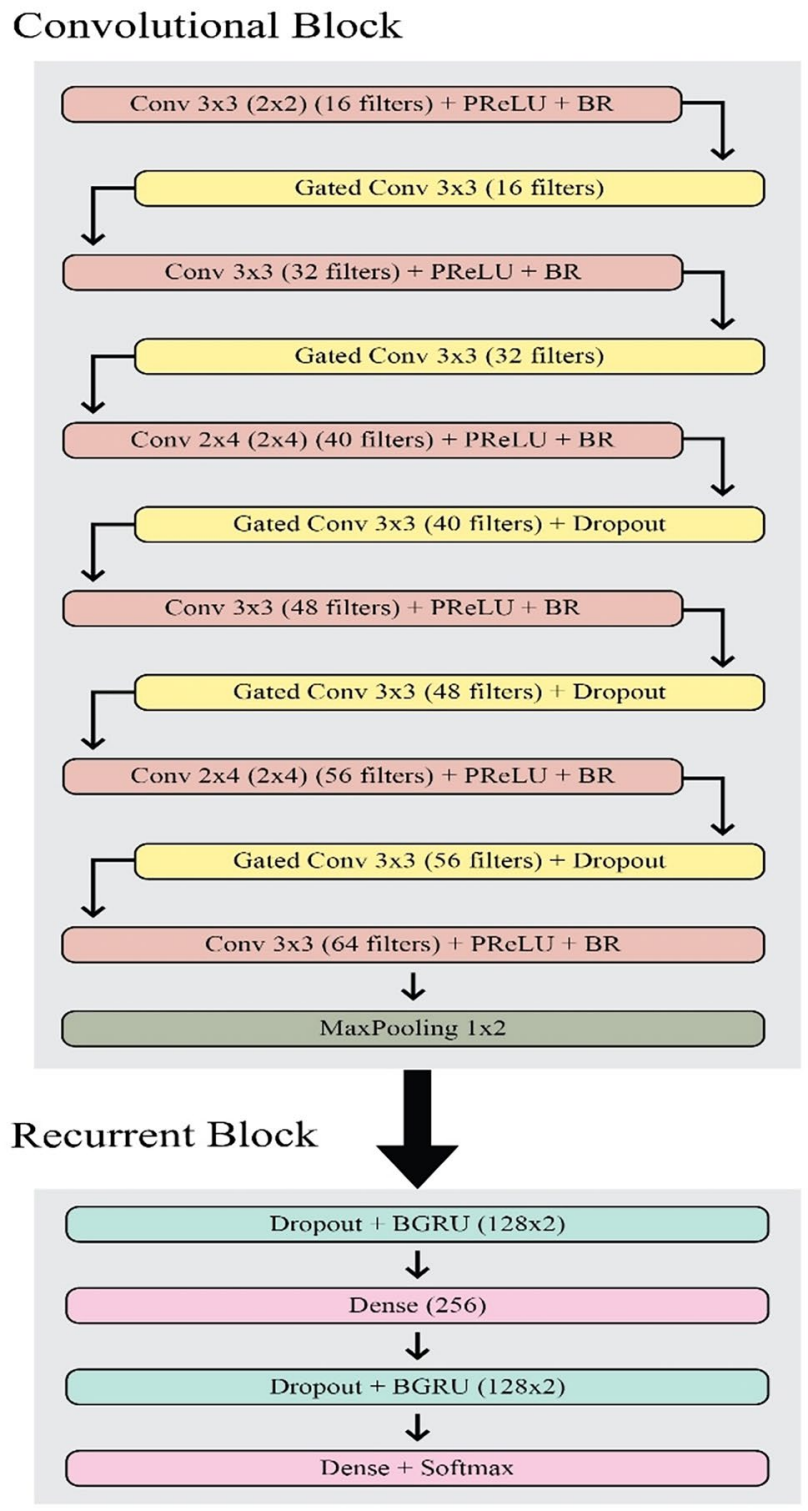
Flor architecture.

The proposed use of a gated mechanism gives better results compared to the Bluche approach because it gives higher performance with a smaller number of parameters (approx. 820,000). The architecture also uses BGRU instead of BLSTM. Figure 3 depicts the architecture presented by Flor which includes 6 convolutional layers, 5 gated convolutional layers, and 2 BGRU.

The convolutional block contains 11 layers of which 6 are traditional ones and the remaining five are gated convolutional layers. The block can be divided into 7 parts i.e. (i) combination of gated convolutional layer of 3 × 3 kernels and a convolutional layer of 3 × 3 kernels with 16 filters each; (ii) combination of gated convolutional layer of 3 × 3 kernels and a convolutional layer of 3 × 3 kernels with 32 filters each; (iii) combination of gated convolutional layer of 3 × 3 kernels and a convolutional layer of 2 × 4 kernels with 40 filters each; (iv) combination of gated convolutional layer of 3 × 3 kernels and a convolutional layer of 3 × 3 kernels with 48 filters each; (v) combination of gated convolutional layer of 3 × 3 kernels and a convolutional layer of 2 × 4 kernels with 56 filters each; (vi) convolutional layer of 3 × 3 kernels with 64 filters, and (vii) to overcome overfitting Max pooling is applied with 1 × 2 kernels. The role of initiator is played by He uniform rather than Glorot uniform. Parametric Rectifier Linear Unit (PReLU)^22^ is used as an activator. Batch renormalization^23^ is used for nonlinear activation functions. Dropout (probability 0.2) for the last three gated convolutional layers of the convolutional block. LSTM cells use gates to control the flow of information through the network. Here are the equations that describe the functioning of an LSTM cell:

Forget Gate (f_t_):

This gate determines what information from the previous cell state (C _{t-1}_) should be forgotten or retained.

3 $${\text{f}}_{{\text{t}}} = \, \sigma \left( {{\text{W}}_{{\text{f}}} * \, \left[ {{\text{h}}_{{\{ {\text{t}} - {1}\} }} ,{\text{ x}}_{{\text{t}}} } \right] \, + {\text{ b}}_{{\text{f}}} } \right)$$

Input Gate (i_t_):

This gate decides what new information should be stored in the cell state.

4 $${\text{i}}_{{\text{t}}} = \, \sigma \left( {{\text{W}}_{{\text{i}}} * \, \left[ {{\text{h}}_{{\{ {\text{t}} - {1}\} }} ,{\text{ x}}_{{\text{t}}} } \right] \, + {\text{ b}}_{{\text{i}}} } \right)$$

Candidate Cell State (C ~ t):

This is the new candidate value for the cell state.

5 $${\text{C}}_{{\text{t}}} = {\text{tanh}}\left( {{\text{W}}_{{\text{c}}} *{\mkern 1mu} \left[ {{\text{h}}_{{\left\{ {{\text{t}} - 1} \right\}}} ,{\text{x}}_{{\text{t}}} } \right]{\mkern 1mu} + {\text{b}}_{{\text{c}}} } \right)$$

Update Cell State (C_t_):

This equation combines the previous cell state, the forget gate output, and the input gate output to update the cell state.

6 $${\text{C}}_{{\text{t}}} = {\text{ f}}_{{\text{t}}} *{\text{ C}}_{{\{ {\text{t}} - {1}\} }} + {\text{ i}}_{{\text{t}}} *{\text{ C}}\sim_{{\text{t}}}$$

Output Gate (o_t_):

This gate determines what part of the cell state should be output as the hidden state.

7 $${\text{o}}_{{\text{t}}} = \, \sigma \left( {{\text{W}}_{{\text{o}}} * \, \left[ {{\text{h}}_{{\{ {\text{t}} - {1}\} }} ,{\text{ x}}_{{\text{t}}} } \right] \, + {\text{ b}}_{{\text{o}}} } \right)$$

Hidden State (h_t_):

The hidden state is the output of the LSTM cell and carries relevant information for the next time step.

8 $${\text{h}}_{{\text{t}}} = {\text{ o}}_{{\text{t}}} *{\text{ tanh}}\left( {{\text{C}}_{{\text{t}}} } \right)$$

Figure 3 depicts the Flor Architecture, a well-known model in the field of Handwritten Text Recognition (HTR) developed by Flor and praised for its creative design and performance. This architecture is deliberately designed to address the complexities of recognising handwritten text, especially in environments with various handwriting styles and adverse environmental conditions. The Flor Architecture is often made up of a set of interconnected modules that are painstakingly designed to capture key elements from raw input, model sequential dependencies, and produce accurate text predictions. Core components may include convolutional neural network (CNN) layers for robust feature extraction, recurrent neural network (RNN) layers like Long Short-Term Memory (LSTM) or Gated Recurrent Units (GRU) for capturing sequential patterns, and attention mechanisms for dynamically focusing on relevant regions of the input. The Flor Architecture performs admirably in handwritten text recognition tasks across a variety of datasets and real-world applications by managing these components in a unified manner. Furthermore, this design acts as a baseline for assessing the efficacy of novel HTR procedures and techniques, providing vital insights that will push future improvements in the field.

The recurrent block consists of 4 layers i.e. (i) is a BGRU layer with 127 hidden units with a dropout (probability 0.5); (ii) is a dense layer consisting of 256 hidden units; (iii) is a BGRU layer with 128 hidden units with a dropout (probability 0.5); and (iv) is a dense layer as the last layer of the recurrent block with a size of 1 (CTC null symbol) + charset size.

## Proposed model

**Figure 4:**
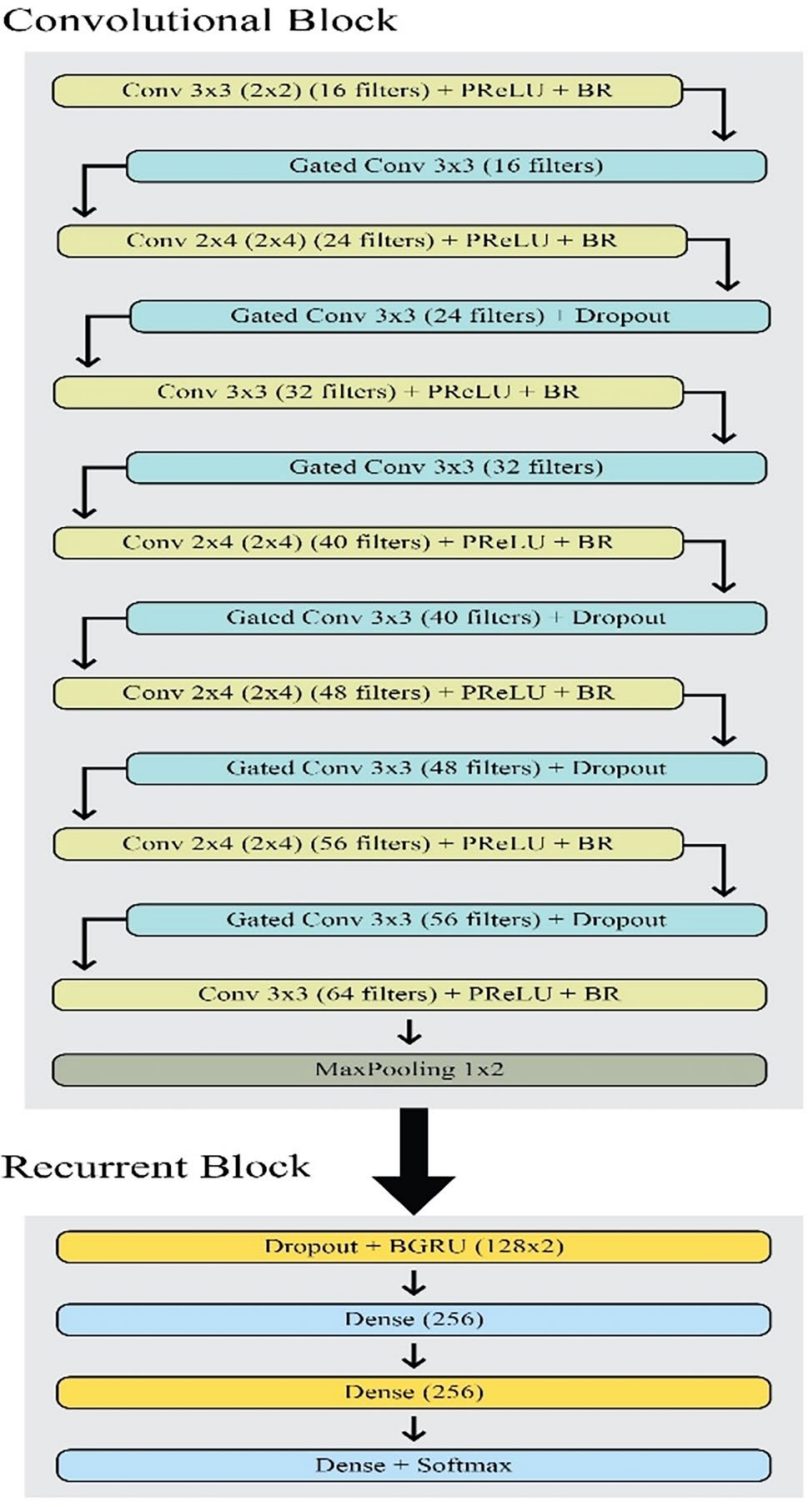
Proposed architecture.

The Puigcerver, Bluche, and Flor models served as inspiration for the proposed model. In order to improve the model's accuracy with fewer parameters (roughly 830,000), it uses a Gated mechanism and architecture that is like Flor with a few minor modifications^15^. Figure 4 shows the proposed architecture, which consists of 2 BGRU, 6 gated convolutional layers, and 7 convolutional layers.

The convolutional block consists of 13 layers of which 7 are traditional ones and the remaining six are gated convolutional layers. The block can be divided into 8 parts i.e. (i) combination of gated convolutional layer of 3 × 3 kernels and a convolutional layer of 3 × 3 kernels with 16 filters each; (ii) combination of gated convolutional layer of 3 × 3 kernels and a convolutional layer of 2 × 4 kernels with 24 filters each; (iii) combination of gated convolutional layer of 3 × 3 kernels and a convolutional layer of 3 × 3 kernels with 32 filters each; (iv) combination of gated convolutional layer of 3 × 3 kernels and a convolutional layer of 2 × 4 kernels with 40 filters each; (v) combination of gated convolutional layer of 3 × 3 kernels and a convolutional layer of 3 × 3 kernels with 48 filters each; (vi) combination of gated convolutional layer of 3 × 3 kernels and a convolutional layer of 2 × 4 kernels with 56 filters each; (vii) convolutional layer of 3 × 3 kernels with 64 filters, and (viii) to overcome overfitting Max pooling is applied with 1 × 2 kernels.

The role of initiator is played by He uniform rather than Glorot uniform. Parametric Rectifier Linear Unit (PReLU) is used as an activator. Batch renormalization is used for the nonlinear activation function. Dropout (probability 0.2) for the last three gated convolutional layers of the convolutional block.

9 $$G\left( y \right) = \beta \left( {E_{g,y} \overline{c}_{y} + E_{g,y} \overline{j}_{y - 1} + n_{g,y} } \right)$$

In Eq. (9), the term $\beta$ represents the sigmoid function, and the biasing term of the forget gate is denoted by $n_{g,y}$.

10 $$\overline{V}_{t} = \tan \left( {E_{{v,\overline{c}_{y} }} + E_{{v,\overline{c}_{y - 1} }} + \overline{n}_{g,y} } \right)$$

11 $$V_{y} = g_{y} \cdot V_{y - 1} + o_{y} \cdot \overline{V}_{y}$$

12 $$Out = \sigma \left( {E_{p} h\left( {j_{y - 1} ,c_{y} } \right) + n_{p} } \right)$$

In Eqs. (10)–(12), the terms $V_{y}$ and $V_{y - 1}$ denote the current and past status of the memory and $n_{p}$ indicates the biased term.

In this scenario, more straightforward optical models were devised in order to achieve the same or better performance as their more conventional counterparts. Therefore, Puigcerver ^13^ suggested the CNN-BLSTM architecture as a means of reducing the computational cost while simultaneously achieving better results than cutting-edge models that utilised Multidimensional LSTM (MDLSTM) layers. This was done in order to achieve better results. Similarly, Bluche et al^18^. presented an architecture called Gated-CNNBLSTM. This architecture makes use of the Gated mechanism in the convolutional layers as a way to extract more pertinent features. This enables a significant reduction in the number of parameters required by the optical model and achieves impressive results.

The recurrent block consists of 4 layers i.e. (i) is a BGRU layer with 128 hidden units with a dropout (probability 0.5); (ii) is a dense layer consisting of 256 hidden units; (iii) is a BGRU layer with 128 hidden units with a dropout (probability 0.5); and (iv) is a dense layer as the last layer of the recurrent block with a size of 1 (CTC null symbol) + charset size.

## Datasets and methods

To assess the performance of the proposed models against recognised benchmarks, extensive testing was carried out on renowned datasets, including Puigcerver (13), Bluche (18), and Flor (19). These datasets are widely recognised in the Handwritten Text Recognition (HTR) field and are used as standard benchmarks for determining recognition accuracy and robustness. To allow for rigorous training, validation, and testing, each dataset was methodically separated into three separate groups. Specifically, the Bentham, RIMES, IAM, Washington, and Saint Gall datasets used partitioning algorithms that were adapted to their specific datasets. The first table describes the partitioning approach for text line picture data, which ensures that the evaluation procedure is consistent and reliable. This comprehensive approach ensures a complete evaluation of the suggested models' performance against established benchmarks, yielding significant insights into their usefulness and potential for advancement in the field of HTR.

These models were tested against the well-known datasets Puigcerver (13), Bluche (18), and Flor (19) in order to see how well they performed compared to the proposed model.

### Datasets

For training, validation, and testing, all of the datasets have been divided into three categories. The Bentham, RIMES, IAM, Washington, and Saint Gall datasets each have their own unique partitioning strategy for storing their data. Text line image partitioning is shown in the Table 1.

**Table 1:** Description of Datasets.

Dataset	Fragmentation			Run-of-the-mill tokens/Decree		Length of the Decree		Total
	Analysis	Keeping fit	Authentication	Words	Characters	Maxima	Minima	
RIMES	780	10,189	1129	44	9	111	3	12,265
Washington	159	330	170	43	8	60	5	775
IAM	1859	6159	899	48	9	80	7	9061
Bentham	859	9201	1409	46	8	106	3	11,632
Saint Gall	710	470	240	57	8	76	7	1568

#### Bentham

**Figure 5:**
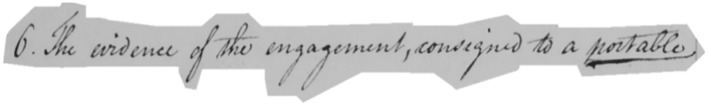
Bentham database sample.

Jeremy Bentham^3^, an English philosopher, is the author of the dataset. The Bentham dataset consists of several historical manuscripts that have been converted into grayscale images with obtrusive text and dark backgrounds as represented in Fig. 5. There are about 11,630 text lines in this dataset. The 9195 training, 1410 validation, and 859 testing images make up the partitioning subsets. The main issue with this dataset is how many punctuation marks there are in the text lines.

#### IAM

**Figure 6:**
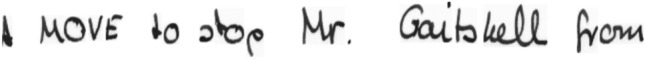
IAM database sample.

The IAM dataset contains 1539 handwritten English text pages that were scanned in grayscale as represented in Fig. 6. The 9000 outlines of text in the IAM dataset were penned by 657 different authors. The outlines transcribed by one writer belong to a single subset because the dataset was created for HTR systems to be independent of the writer's handwriting. The 6159 training, 899 validation, and 1859 test images make up the partitioning subsets. The main issue with this dataset is the sheer volume of writers, and some of the images have very difficult-to-recognize cursive handwriting.

#### RIMES

**Figure 7:**
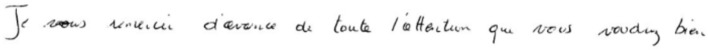
RIMES database sample.

The 12,000 handwritten lines in the RIMES dataset were taken from 5600 French-language emails as represented in Fig. 7. The text is easier to read and the background is more transparent in the images of the text lines. The dataset was designed for HTR systems to be independent of the writer's handwriting, therefore the text lines produced by a single writer belong to a single subset. The 6161 training, 900 validation, and 1861 test images make up the partitioning subsets. Most of the words in this dataset are based on local dialects, which presents a challenge.

#### Saint Gall

**Figure 8:**
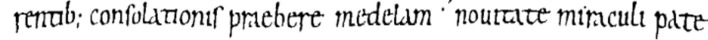
Saint Gall database sample.

The dataset was written in the ninth century by a Latin speaker. The Saint Gall dataset is a group of Latin-language ancient documents as represented in Fig. 8. 48 unique characters and about 6000 unique words make up this dataset. There are roughly 1410 text lines in this dataset. The 468 images used for training, the 235 images used for confirmation, and the 707 images used for testing make up the partitioning subsets. This dataset has the benefit of having normalized and binarized text line images. The main issue with this dataset is that there is a very small amount of data, which could lead to overfitting.

#### Washington

**Figure 9:**
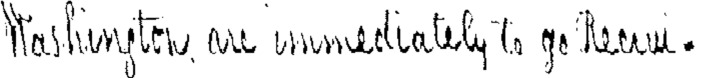
Washington database sample.

By means of papers written by George Washington in the eighteenth century, a dataset was created Historic manuscripts by two authors are included in the Washington dataset as represented in Fig. 9, which has less data than Saint Gall. About 1189 distinct words and 68 distinct characters make up this collection. The text in this dataset is approximately 656 lines long. 325 training images, 168 validation images, and 163 testing images make up the partitioning subsets. The text line images in this dataset have already been normalized and binarized, which is an advantage. Overfitting is a major problem with this dataset because it has a small amount of data.

#### Exploratory setup

The Puigcerver archetypal used imagery of entire paragraphs as hyper parameters for each case. Blucher’s model was trained with 132,000 images from a large private set. Flor's model made use of images of text lines. Therefore, we will expend the same workflow and hyperparameters for all datasets and models in order to ensure that the statistical results are comparable. This concept was motivated by the work of^10^.

The investigational setup begins by preparing the optical models and CTC functions in order to increase the loss value. The RMS prop optimizer^24,25^ is used with a mini-batch of 16 images and a learning rate of 0.001 in each step. In order to enhance the loss value, the learning rate is decreased by a factor of 0.2 after 15 iterations in which there is no improvement, and early halting is used after 20 iterations. For the CTC, we have used^26^ Word Beam Search. There are 150 characters in the ASCII table that can be used for encoding and decoding.

To understand the model better, the project's images must be normalized. In order to balance brightness and contrast, illumination compensation^27–29^ is used to normalize all the images. (ii) Desalting the images of cursive writing from^30,31^. (iii) All images are resized and padded to 1024 × 128 × 1. (iv) For all input images, data expansion, such as movement change and morphological ascending, is carried out in three steps, namely (a) rotation and scaling by 30 and 5 percent, respectively. (b) A 5% change in both height and width. (c) Up to 5 × 5 kernels and 3 × 3 kernels, respectively, of erosion and dilation. N-gram statistical characters were used to improve the results. SRILM Toolkit, a free software programme, is used with language models^28,32^. The language model can be easily trained because it uses text rather than images^33,34^. For running all of the project files using GPU for more powerful computational power, the project uses Google Colaboratory, another free to use online simulator^35–37^.

### Exploratory evaluation

Character Error Rate and Word Error Rate are the two metrics used to experimentally evaluate the models. The Levenshtein distance^29^ between the predictions and the truth is used to calculate them. We need a *p*-value that is less than alpha, or 0.05, in order to say that the proposed model has a lower error rate.

## Results and discussion

The models were applied to a variety of well-known datasets in an effort to improve upon the performance of prior models such as Puigcerver, Bluche, and Flor. These datasets include Washington, Bentham, RIMES, Saint Gall, and IAM. The CER and WER values that can be derived by utilising our suggested model have a *p*-value that is less than 0.01 when compared to the models that have already been declared. Each model's *p*-values have been drastically reduced, and those new values may be found in the tables that relate to each dataset.

The char 9-g language model is the one that is utilised for the Bentham dataset. In this particular dataset, the loss per word that can be attributed to punctuation marks is 25%. Both the CER and WER for the model under consideration come in at 2.71% and 8.50%, respectively. When compared to other models, such as Puigcerver, Bluche, and Flor by, the WER shows a considerable improvement. The improvements come in the form of 3.79%, 8.50%, and 1.29% correspondingly. Table 2 contains a discussion of the findings for both the with and without punctuation marks cases.

**Table 2:** WER and CER for Bentham Test Partition.

Optical Model + char 9-g	Only Confrontations		Chock-full Text	
	WER	CER	WER	CER
Bluche	15.53%(± 0.24)	6.69%(± 0.10)	13.8% ± 0.19)	5.81%(± 0.05)
Flor	9.69%(± 0.20)	4.01%(± 0.04)	6.7%(± 0.15)	3.4%(± 0.03)
Puigcerver	11.99%(± 0.21)	4.70%(± 0.04)	9.10%(± 0.13)	3.98%(± 0.04)
Suggested Model	8.50%(± 0.12)	2.71%(± 0.04)	3.79%(± 0.13)	3.01%(± 0.03)

	
Puigcerver			Bluche	
	
Flor			Proposed Model	

For the IAM dataset, the char 8-g language model is used. The loss per word due to punctuation marks in this dataset is 2%. The CER and WER for the proposed model are 2.41% and 9.79% respectively. So, when compared with other models, WER is significantly improved compared to Puigcerver, Bluche, and Flor by 3.88%, 8.04%, and 1.34% respectively. Table 3 contains a discussion of the findings for both the with and without punctuation marks cases.

**Table 3:** WER and CER for IAM Test Partition.

Optical Model + char 9-g	Only confrontations		Chock-full text	
	WER	CER	WER	CER
Bluche	17.58%(± 0.21)	6.09%(± 0.10)	17.91%(± 0.14)	6.59%(± 0.08)
Flor	10.89%(± 0.18)	3.29%(± 0.07)	11.21%(± 0.09)	3.68%(± 0.08)
Puigcerver	12.21%(± 0.19)	4.29%(± 0.06)	13.69%(± 0.16)	4.89%(± 0.09)
Suggested Model	6.28%(± 0.16)	2.69%(± 0.05)	9.79%(± 0.17)	2.41%(± 0.04)

	
Puigcerver			Bluche	
	
Flor			Proposed Model	

For the RIMES dataset, the char 12-g language model is used. The loss per word due to punctuation marks in this dataset is 14%. The CER and WER for the proposed model are 2.7% and 10.2% respectively. So, when compared with other models, WER is significantly improved compared to Puigcerver, Bluche, and Flor by 1.49%, 4.61% and 0.96% respectively. Table 4 contains a discussion of the findings for both the with and without punctuation marks cases.

**Table 4:** WER and CER for RIMES Test Partition.

Optical Model + char 9-g	Only Confrontations		Chock-full Text	
	WER	CER	WER	CER
Bluche	14.59%(± 0.18)	4.8%(± 0.10)	14.81%(± 0.20)	5.19%(± 0.09)
Flor	8.69%(± 0.20)	2.64%(± 0.05)	11.16%(± 0.21)	3.3%(± 0.08)
Puigcerver	9.9%(± 0.20)	3.21%(± 0.08)	11.7%(± 0.21)	3.69%(± 0.11)
Suggested Model	8.7%(± 0.18)	1.81%(± 0.04)	10.2%(± 0.17)	2.7%(± 0.07)

	
Puigcerver			Bluche	
	
Flor			Proposed Model	

For the Saint Gall dataset, the char 11-g language model is used. The loss per word due to the punctuation mark in this dataset is zero. The CER and WER for the proposed model are 3.81% and 18.61% respectively. So, when compared with other models, WER is significantly improved compared to Puigcerver, Bluche, and Flor by 5.07%, 5.10%, and 2.51% respectively. Table 5 contains a discussion of the findings for both the with and without punctuation marks cases.

**Table 5:** WER and CER for Saint Gall Test Partition.

Optical Model + char 9-g	Only Confrontations		Chock-full Text	
	WER	CER	WER	CER
Bluche	23.69%(± 0.12)	5.99%(± 0.06)	23.71%(± 0.12)	5.99%(± 0.05)
Flor	21.11%(± 0.12)	5.25%(± 0.02)	21.11%(± 0.11)	5.27%(± 0.04)
Puigcerver	23.4%(± 0.02)	5.97%(± 0.04)	23.69%(± 0.02)	5.89%(± 0.06)
Suggested Model	18.61%(± 0.13)	3.81%(± 0.03)	18.61%(± 0.10)	3.88%(± 0.03)

	
Puigcerver			Bluche	
	
Flor			Proposed Model	

For the Washington dataset, the char 10-g language model is used. The loss per word due to punctuation marks in this dataset is 3%. The CER and WER for the proposed model are 2.97% and 7.55% respectively. So, when compared with other models, WER is significantly improved compared to Puigcerver and Bluche by 25.36% and 14.42% respectively. But the results are comparable with the results of the Flor model. Table 6 contains a discussion of the findings for both the with and without punctuation marks cases.

**Table 6:** WER and CER for Washington Test Partition.

Optical Model + char 9-g	Only Confrontations		Chock-full Text	
	WER	CER	WER	CER
Bluche	21.30%(± 0.2)	10.4%(± 0.09)	22.0%(± 0.16)	10.89%(± 0.13)
Flor	7.60%(± 0.09)	2.57%(± 0.05)	7.9%(± 0.13)	2.99%(± 0.03)
Puigcerver	34.3%(± 0.18)	18.69%(± 0.14)	32.89%(± 0.19)	19.3%(± 0.12)
Suggested Model	6.6%(± 0.09)	2.58%(± 0.03)	7.55%(± 0.15)	2.97%(± 0.03)

	
Puigcerver			Bluche	
	
Flor			Proposed Model	

### The results

For the All-in-one dataset, the CER and WER for the proposed model are 2.89% and 10.89%, respectively. Compared with other models, WER is significantly improved compared to Puigcerver, Bluche, and Flor by 7.88%, 8.10%, and 1.28%, respectively. Table 7 contains a discussion of the findings for both the with and without punctuation marks cases.

**Table 7:** WER and CER evaluation for all Test Partition.

Optical Model + char 9-g	Only confrontations		Chock-full text	
	WER (%)	CER (%)	WER (%)	CER (%)
Bluche	18.19	6.59	18.99	7.11
Flor	11.12	3.51	12.33	3.91
Puigcerver	17.8	7.19	18.91	7.69
Suggested Model	8.79	2.82	10.89	2.89

**Figure 10:**
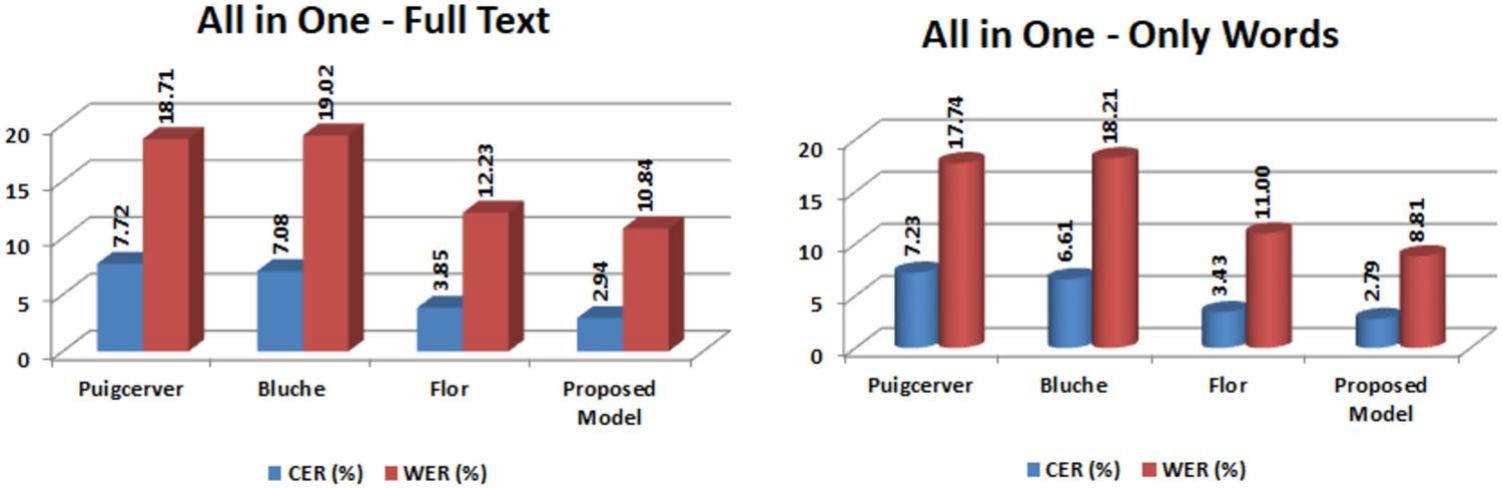
WER and CER evaluation for all Test partition.

Figure 10 shows the graphical comparison of WER and CER evaluation for all test partition. The performance of the recommended ideal is superior to that of the existing mockups for three reasons: I the use of cutting-edge deep learning methods and tool-kits; (ii) the use of a convolutional block gated mechanism; and (iii) the use of bidirectional gated recurring parts in the regular block. According to the results, the performance was enhanced in comparison to all of the earlier introduced models; however, the suggested parameters are only inferior to the Puigcerver prototypical and higher than both the Flor and Bluche models. This is despite the fact that the performance was improved in comparison to all of the previously introduced models.

The performance of the suggested models was rigorously evaluated across numerous well-known datasets, including Puigcerver, Bluche, and Flor, to determine their superiority over existing approaches. These datasets, which include Washington, Bentham, RIMES, Saint Gall, and IAM, were rigorously partitioned for training, validation, and testing to ensure rigorous evaluation. Statistical evaluations demonstrated considerable improvements in Character Error Rate (CER) and Word Error Rate (WER) values over known models, with p-values less than 0.01. For example, using the Bentham dataset, the suggested model outperformed Puigcerver, Bluche, and Flor in terms of WER by 3.79%, 8.50%, and 1.29%, respectively. Similar trends were seen in other datasets, demonstrating the effectiveness of the proposed approach. Notably, graphical comparisons in Fig. 10 show that the recommended model outperforms all test partitions, owing to its use of cutting-edge deep learning methodologies, convolutional block gated mechanisms, and bidirectional gated recurrent components. While the suggested parameters fall somewhat behind the Puigcerver prototype, they outperform both the Flor and Bluche models, indicating a significant improvement over previous techniques. Overall, these findings demonstrate the suggested models' great potential for expanding the field of Handwritten Text Recognition, opening the way for future research efforts.

## Conclusion

In this paper, enhanced Flor's Gated-CNN-BGRU was used, which was followed by two steps of language processing to produce outcomes resembling handwritten images. In conclusion, our work offered innovative models aimed at improving the performance of existing Handwritten Text Recognition (HTR) systems, especially when compared to established models such as Puigcerver, Bluche, and Flor. These models were thoroughly tested across a variety of datasets, including Washington, Bentham, RIMES, Saint Gall, IAM, and an all-in-one dataset. Following extensive examination, our proposed models consistently displayed improved performance, as proven by much reduced Character Error Rate (CER) and Word Error Rate (WER) values. Across all datasets, the WER improvements over Puigcerver, Bluche, and Flor ranged from 0.96% to 8.10%.

Furthermore, statistical studies indicated much lower p-values, suggesting the strength of our models' performance when compared to existing ones. Furthermore, our models demonstrated advances in managing punctuation marks, with considerable improvements in datasets where punctuation was important. For example, in the Bentham dataset, where punctuation accounts for 25% of the loss per word, our model outperformed earlier models in terms of WER.

Our future research will focus on HTR and novel strategies for improving model accuracy, speed, and flexibility. We wish to investigate how sophisticated attention processes can aid models in acquiring contextual information, particularly in complex handwriting styles and noisy input data. We also wish to look into domain adaptation approaches to allow for smooth generalization across different datasets and real-world scenarios, as well as to make our models more robust to writing styles and environmental variables. To ensure that our solutions are practical and effective across application domains, we will design user-centric HTR systems based on user feedback and usability studies. We aim to promote HTR research by developing more accurate, efficient, and user-friendly handwritten text recognition systems through interdisciplinary collaboration and innovation.

## Data Availability

The datasets used during the current study are available from the corresponding author on reasonable request.
